# *Giardia intestinalis* in Thailand: Identification of Genotypes

**DOI:** 10.3329/jhpn.v28i1.4522

**Published:** 2010-02

**Authors:** Anchalee Tungtrongchitr, Nitat Sookrung, Nitaya Indrawattana, Sukanya Kwangsi, Jeerawan Ongrotchanakun, Wanpen Chaicumpa

**Affiliations:** ^1^ Department of Parasitology; ^2^ Office for Research and Development, Faculty of Medicine Siriraj Hospital, Mahidol University, Bangkok 10700, Thailand; ^3^ Department of Microbiology and Immunology, Faculty of Tropical Medicine, Mahidol University, Bangkok 10400, Thailand

**Keywords:** β-*giardin*, Genotyping, *Giardia duodenalis*, *Giardia intestinalis*, Giardiasis, Glutamate dehydrogenase, Triose phosphate isomerase, Thailand

## Abstract

This study was undertaken to determine the genetic diversities of *Giardia intestinalis* isolated in Thailand. *G. intestinalis* cysts were collected from stool samples of 61 subjects residing in Bangkok or in rural communities of Thailand with and without gastrointestinal symptoms. All the cyst samples gave positive *tpi* amplicons (100% sensitivity), either of the 148- or the 81-bp *tpi* segments. Cyst assemblage identification of the 148- and 81-bp *tpi* gene segments by polymerase chain reaction showed that 8% of the cysts were assemblage A, 41% assemblage A and B combined, and 51% assemblage B. The prevalence of assemblage A was significantly lower than that of assemblage B and the mixed types. Restriction fragment length polymorphism (RFLP) of the 384-bp β-*giardin* gene segment revealed that 12% and 88% of the assemblage A cysts were AI and AII respectively. RFLP, based on the 432-bp *gdh* gene segment, showed 45.5% of the assemblage B cysts to be BIII and 54.5% to be BIV. The AI sub-assemblage was less prevalent than the others. All subjects with AI and 50% of the subjects with BIII sub-assemblage cysts were symptomatic; 80% of symptomatic Bangkok residents were adults/elderly while 85% of the rural cases were children.

## INTRODUCTION

Flagellated protozoa of the genus *Giardia* comprise several species and are ubiquitous. The parasite infects the intestinal tract of a wide range of vertebrate hosts belonging to different phyla, including avian, reptile, mammal, and human (1–3). Infection in humans is caused by *Giardia intestinalis* (synonyms: *G. lamblia, G. duodenalis*), which is molecularly classified into seven genotypes (assemblages), namely A-G (4–5). Humans are mainly infected by genotype A and/or B (6). *Giardia*-associated infection is the most common intestinal infestation worldwide (7). The worldwide incidence was estimated at 2.8×10^8^ infections per year (8). Humans are infected either by faecal-oral transmission through fomites or by ingestion of parasite cysts in contaminated drinking-water and less often in food (9). Excystation of the gastric acid-stimulated cysts occurs in the host intestine, and the trophozoites subsequently infect the intestinal mucosa, multiply, and colonize the mucosal surface, thus, interfering with the host's nutrient absorption (10). Although 60–80% of *G. intestinalis*-associated infections are asymptomatic, clinical manifestations ranging from mild to severe diarrhoea with bloating and abdominal pain are not infrequent, especially among young children and the elderly (9, 10). Chronic giardiasis as well may cause long-term growth retardation due to nutritional deficiencies (11).

In Thailand, the prevalence of human giardiasis ranged from 1.25% to 37.7% (12). The incidence varies depending on age, living circumstances, environmental sanitation, and personal hygiene. *G. intestinalis* is a common parasite in Thailand and in other developing countries (12–16). The incidence among Thai orphans is 37.7–85.5% (17, 18). Nevertheless, this parasitic infestation did not receive much attention or concern so far. In this study, *G. intestinalis* isolates from asymptomatic subjects and from those with gastrointestinal symptoms were genetically typed. The data provide additional molecular information on human *G. intestinalis* isolates which was so far lacking from Thailand.

## MATERIALS AND METHODS

### Human subjects, stool samples, and purification of *G. intestinalis* cysts

Of 6,967 subjects included, 6,018 were Bangkok inhabitants who visited the Siriraj Hospital during December 2006–July 2007 for health check-ups. *G. intestinalis* cysts were microscopically found in stools of 22 subjects (0.36%). Seventeen subjects (10 male, 7 female) were asymptomatic (Group 1), and five subjects (3 male, 2 female) had gastrointestinal symptoms (Group 2), including one or more of the following complaints: (chronic) diarrhoea with either soft, mucoid, or watery stool, and/or abdominal pain. The other 949 subjects were inhabitants of Ratchaburi province (located nearly 100 km southwest of Bangkok) recruited during a community survey conducted in September 2007. *G. intestinalis* cysts were found in 39 subjects (4.1%). Twenty-six (12 male, 14 female) of these were asymptomatic (Group 3), and 13 (5 male, 8 female) complained of intestinal symptoms (Group 4). Background information of the *G. intestinalis*-infected subjects is presented in Table 1.

**Table 1.** Background information on the subjects from whom *G. intestinalis* cysts were collected, the results of gene segment amplifications, and the assemblage/sub-assemblage classification of the cysts

Source	Group	No.	Age (years)	Sex	PCR amplicon				Assemblage(s)*	Sub-assemblage(s)**
					*ssrRNA* (292 bp)	β-*giardin* (384 bp)	β-*giardin* (753 bp)	*gdh* (432 bp)		
Siriraj	1 (healthy)	1	7	F	-	+	+	+	A+B	AII+BIII
Hospital		2	14	M	+	+	+	-	A+B	AII
		3	34	M	-	+	+	+	B	BIV
		4	57	M	+	+	+	+	B	BIV
		5	15	F	-	+	+	+	A+B	AII+BIII
		6	2	M	-	+	+	-	B	
		7	47	F	-	+	-	-	B	
		8	33	F	-	-	-	-	B	
		9	18	F	-	+	-	-	B	
		10	37	M	-	+	+	-	A+B	AII
		11	44	M	+	+	+	-	A+B	AII
		12	32	F	-	-	-	-	B	
		13	9	M	+	+	+	-	A+B	AII
		14	55	M	-	+	-	-	B	
		15	35	M	-	+	-	-	B	
		16	58	F	-	-	-	-	B	
		17	70	M	+	+	+	+	A+B	AII+BIV
	2 (symptomatic)	18	38	F	-	+	+	+	A+B	AII+BIII
		19	50	M	-	+	+	+	A+B	AII+BIV
		20	9	M	+	-	-	-	B	
		21	54	M	+	+	-	-	B	
		22	66	F	+	+	+	+	A+B	AI+BIV
Ratchaburi	3 (healthy)	23	4	M	+	+	+	-	B	
province		24	7	F	-	+	+	-	A	AII
		25	29	M	-	-	-	-	B	
		26	2	F	-	+	+	-	B	
		27	3	F	-	-	-	-	B	
		28	4	F	-	-	-	-	B	
		29	6	F	+	+	+	-	B	
		30	9	M	-	+	-	+	A+B	AII+BIV
		31	11	F	-	+	+	-	A+B	AII
		32	3	M	-	-	-	+	B	BIV
		33	9	M	-	+	-	-	A	AII
		34	12	M	-	+	+	-	B	
		35	10	F	-	+	+	-	B	
		36	31	M	-	+	+	+	B	BIV
		37	43	F	-	+	+	-	A+B	AII
		38	42	M	-	-	-	+	B	BIV
		39	69	F	-	-	-	-	A+B	
		40	6	F	-	+	-	-	B	
		41	5	F	+	+	+	+	A+B	AII+BIII
		42	13	M	-	-	-	+	A+B	BIV
		43	10	M	-	+	+	-	A	AII
		44	4	F	-	+	-	-	A	AII
		45	7	M	-	+	+	-	A+B	AII
		46	13	F	+	+	-	-	B	
		47	14	M	-	-	-	-	A+B	
		48	3	F	-	-	-	-	B	
	4 (symptomatic)	49	5	M	-	-	-	-	B	
		50	3	F	+	+	+	+	A+B	AII+BIII
		51	7	F	-	-	-	-	A+B	
		52	8	F	-	-	-	+	A+B	BIII
		53	2	F	-	-	-	+	B	BIV
		54	4	M	-	+	-	+	B	BIII
		55	7	M	-	+	+	+	A+B	AII+BIII
		56	6	M	-	+	-	+	A+B	AI+BIII
		57	6	M	+	+	-	-	B	
		58	50	F	-	+	-	-	A	AII
		59	4	F	-	+	-	+	A+B	AII+BIII
		60	5	F	-	+	-	+	A+B	AI+BIV
		61	5	F	-	+	+	-	B	
Number of detected/total samples			14/61	44/61	28/61	22/61		

*Assemblage classification was based on the results of *tpi* amplification (assemblage A was positive for the 148-bp *tpi* amplicon, and assemblage B was positive for the 81-bp *tpi* amplicon);

**Sub-assemblages were based on the RFLP of the 384 β-*giardin* and *gdh* amplicons (see text);

PCR=Polymerase chain reaction;

RFLP=Restriction fragment length polymorphism

The *G. intestinalis* cysts were purified from 61 stool samples as previously described with modifications (18). Briefly, 20 g of each sample were stirred in distilled water and filtered through a 70-μm strainer. Aliquots of each preparation were overlaid onto an appropriate volume of a 4-M sucrose solution contained in a tube, and the tube was centrifuged at 850 × g for 10 minutes. The *G. intestinalis* cysts were then collected from the water-sucrose interface, washed several times with normal saline solution, and kept at −70 °C until genomic DNA extraction performed within one month.

### DNA isolation, polymerase chain reaction amplification of gene segments, DNA sequencing, and identification of assemblages

The genomic DNA of the *G. intestinalis* cysts of all the subjects was extracted using a genomic DNA extraction-kit (SBS Gentech, China) according to the instructions of the manufacturer. These DNA preparations, together with the 11 oligonucleotide primer sequences presented in Table 2, were then used for polymerase chain reaction (PCR) amplifications of the 292-bp *ssrRNA* (19), 753-bp and 384-bp β-*giardin* (19), 432-bp *gdh* (20), and 148-bp and 81-bp *tpi* segments which were specific for assemblage A and B respectively (21), under PCR conditions previously described (19–21) using *pfu* DNA polymerase (Fermebtus, Lithuania). The sizes of the DNA amplicons were determined by a 1.0% agarose gel electrophoresis, ethidium bromide staining, and ultraviolet transillumination compared to a 1-kb DNA ladder run concurrently in the same gel slab. The DNA sequences of each PCR amplicon were verified using the ABI Prism BigDye Terminator Cycle Sequencing kit (Applied Biosystems, USA). The DNA sequences of randomly-selected cysts of subjects with symptomatic giardiasis belonging to different assemblages/sub-assemblages were aligned with the database sequences.

**Table 2.** Oligonucleotide primers used in this study

Gene	Primer	Sequence	Amplicon size (bp)	Reference
*ssrRNA*	F-H11	5-CATCCGGTCGATCCTGCC-3	292	19
	R-RH4	5-AGTCGAACCCTGATTCTCCGCCCAGG-3		
384-bp β-*giardin*	F-376	5-CATAACGACGCCATCGCGGCTCTCAGGAA-3	384	19
	R-G759	5-GAGGCCGCCCTGGATCTTCGAGACGAC-3		
753-bp β-*giardin*	F-G7	5-AAGCCCGACGACCTCACCCGCAGTGC-3	753	19
	R-G759	5-GAGGCCGCCCTGGATCTTCGAGACGAC-3		
*gdh*-primary PCR	F-gdhe	5-TCAACGTYAAYCGYGGYTTCCGT-3		
	R-gdhi	5-GTTRTCCTTGCACATCTCC-3		
*gdh*-secondary PCR	F-gdhi	5-CAGTACAACTCYGCTCTCGG-3	432	20
	R-gdhe	5-GTTRTCCTTGCACATCTCC-3		
*tpi*-A	F-tpiA	5-GGAGACCGACGAGCAAAGC-3	148	21
	R-tpiA	5-CTTGCCAAGCGCCTCAA-3		
*tpi*-B	F-tpiB	5-AATAGCAGCACARAACGTGTATCTG-3’	81	21
	R-tpiB	5-CCCATGTCCAGCAGCATCT-3		

PCR=Polymerase chain reaction

### Restriction fragment length polymorphism for determining *G. intestinalis* sub-assemblages

For the determination of the A sub-assemblages, the PCR product of the 384-bp β-*giardin* was digested with *Hha*I restriction endonuclease (19). The amplicon of the 432-bp *gdh* was digested with *Rsa*I for the determination of the B sub-assemblages (20). Each digested DNA preparation was subjected to a 12.5% acrylamide gel electrophoresis and ethidium bromide staining. A low-range molecular weight DNA ladder (Fermentas) was included as a size marker in each gel slab. The banding patterns of the DNA restriction fragments were recorded using a gel documentation system (Gel Doc 2000, BioRad, USA) and observed visually. The accuracy of the sub-assemblage classification was verified by aligning the nucleotide sequences with the respective sub-assemblage sequences of the database using the computer software package Clustal_X 1.83 (22).

### Statistical analysis

The Epi Info software (version 6) was used for analyzing data. Comparison of the prevalence of different *G. intestinalis* assemblages in subjects with symptomatic giardiasis and in the asymptomatic subjects and the prevalence of each assemblage between the symptomatic and the asymptomatic subjects was made using chi-square test. A p value of <0.05 was considered statistically significant.

The Ethics Committee of the Faculty of Medicine, Siriraj Hospital, Mahidol University, Bangkok, Thailand, approved the study.

## RESULTS

### PCR for detection of *G. intestinalis* genes

**Fig. 1.**
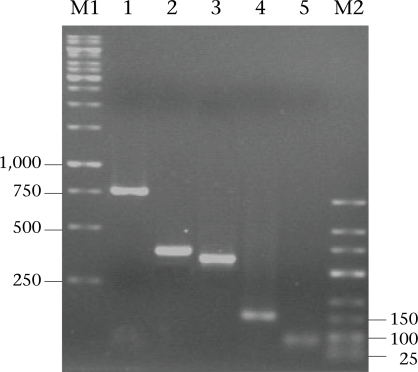
PCR amplicons of β-*giardin, ssrRNA, gdh,* and *tpi* segments

The 292-bp *ssrRNA*, 753-bp β-*giardin*, 384-bp β-*giardin*, and 432-bp *gdh* segments could be amplified from 14 (23%), 28 (46%), 44 (72%), and 22 (36%) of the 61 cyst preparations respectively. Nevertheless, all the 61 samples gave positive *tpi* amplicons (100% sensitivity), either of the 148- or the 81-bp gene segments. Figure 1 shows the representative amplicons of the individual gene segments. The results of the PCR amplifications of the gene segments of all the cyst preparations are shown in Table 1.

Lane M1 and M2: 1 kb and 100-bp DNA ladders respectively;
lane 1: 292-bp amplicon of *ssrRNA* segments;
lane 2 and 3: 384 and 753-bp amplicons of β-*giardin* segments respectively;
lane 4: 432-bp *gdh* gene segments;
and lane 5 and 6: 148-bp (assemblage A) and 81-bp (assemblage B) amplicons of *tpi* segments respectively;
PCR=Polymerase chain reaction

### Assemblage identification

Five (8%) of the 61 cyst preparations were positive for the 148 bp-*tpi* amplicon only, implying assemblage A for these *G. intestinalis* cysts, and 31 (51%) were positive for the 81-bp *tpi* amplicon only, implying assemblage B. The remaining 25 samples (41%) were positive for both *tpi* gene amplicons, indicating the presence of a mixed population of these two assemblages (Table 1). Of the 18 symptomatic subjects (5 of Group 2 and 13 of Group 4), one (5.6%) had assemblage A, seven (38.8%) had assemblage B, and 10 (55.6%) had the mixture (Table 3). Of the 43 asymptomatic individuals, four (9.3%) had assemblage A, 24 (55.8%) had assemblage B, and 15 (34.9%) had both (Table 3). The prevalence of assemblage A in both symptomatic and asymptomatic subjects was significantly lower than that of the B and A+B assemblage(s) (p<0.05%) but the prevalence of B and A+B was not significantly different (p>0.05). There was no significant difference in the prevalence of the individual *G. intestinalis* assemblages between the symptomatic and the asymptomatic subjects (p>0.05) (Table 3).

**Table 3.** Prevalence of *Giardia intestinalis* cyst assemblage(s)

*Giardia intestinalis* genotype	Symptomatic (n=18)		Asymptomatic (n=43)	
	No. %	No. %
A	1	5.6*	4	9.3*
B	7	38.8**	24	55.8**
Mixed types	10	55.6**	15	34.9**

Entries with different superscripts (*,**) are statistically different at p<0.05

### Sub-assemblage classification

**Fig. 2.**
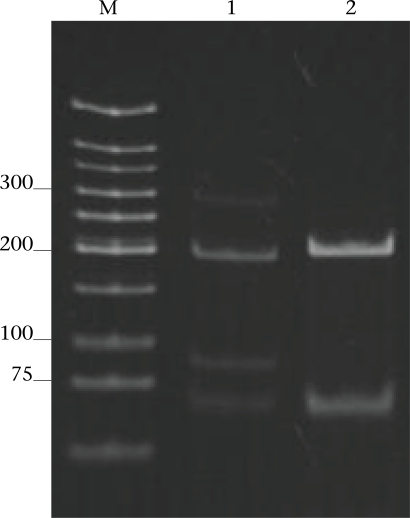
DNA-banding patterns (RFLP) of the 384-bp β-*giardin* gene amplicons cut by the *Hha*I restriction endonuclease

Of the 44 subjects whose *G. intestinalis* cysts were positive for the 384 bp β-*giardin* gene amplicon, 25 were positive for the 148-bp *tpi* amplicon, i.e. assemblage A cysts (5 subjects had A only, and 20 subjects had A+B) (Table 1). To determine the A sub-assemblages, the 384-bp β-*giardin* amplicons of these samples were cut by *Hha*I restriction endonuclease, and the RFLP patterns of the DNA fragments were studied. Only three (12%) (no. 22 of Group 2 and no. 56 and 60 of Group 4) of the 25 subjects had cysts belonging to sub-assemblage AI, as indicated by the presence of three DNA bands at 193, 100 and 70 bp (lane 1, Fig. 2). The remaining 22 subjects (88%) (no. 1, 2, 5, 10, 11, 13, and 17 of Group 1, no. 18 and 19 of Group 2, no. 24, 30, 31, 33, 37, 41, and 43–45 of Group 3, and no. 50, 55, 58, and 59 of Group 4) had sub-assemblage AII, determined by the presence of two DNA bands at 210 and 70 bp (lane 2, Fig. 2).

Lane M: 100-bp DNA ladder;
Lane 1 and 2: DNA-banding patterns of AI and AII sub-assemblages of *Giardia intestinalis* respectively;
RFLP=Restriction fragment length polymorphism

**Fig. 3.**
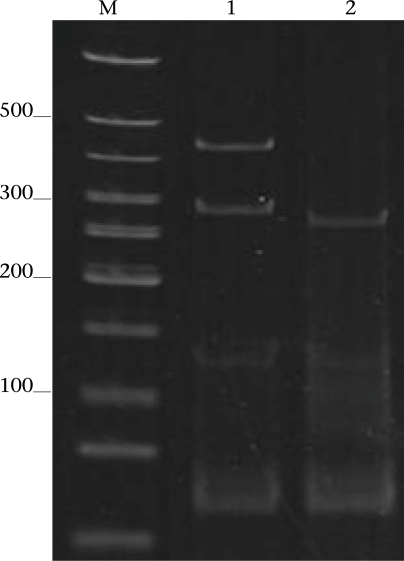
DNA-banding patterns (RFLP) of the 432-bp gdh amplicons cut by the Rsal restriction endonuclease

Of the 56 subjects who had *G. intestinalis* cysts positive for the 81-bp *tpi* segments (31 of assemblage B alone and 25 of assemblages A+B) (Table 1), the 432-bp *gdh* segments could be amplified only from 22, i.e. no. 1, 3–5, and 17 of Group 1, no. 18, 19, and 22 of Group 2, no. 30, 32, 36, 38, 41, and 42 of Group 3, and no. 50, 52–56, 59, and 60 of Group 4 (Table 1). The *gdh* amplicons of these 22 subjects were individually cut by *Rsa*I endonuclease, and the digested products were subjected to agarose gel electrophoresis and ethidium bromide staining for determining the B sub-assemblages. Ten (45.5%) subjects revealed *G. intestinalis* cysts of the BIII sub-assemblage (no. 1, 5, 18, 41, 50, 52, 54–56, and 59), defined by the presence of DNA bands at 290 and 130 bp (lane 1, Fig. 3) while the other 12 subjects (54.5%) harboured cysts of the BIV sub-assemblage, determined by the presence of a 430 DNA band (lane 2, Fig. 3). The distribution of *G. intestinalis* sub-assemblages among the symptomatic and asymptomatic subjects is shown in Table 4. There was no significant difference between the *G. intestinalis* sub-assemblages of the symptomatic and asymptomatic subjects.

**Table 4.** Distribution of *Giardia intestinalis* assemblage(s) and sub-assemblage(s) among symptomatic and asymptomatic Thai subjects

*Giardia intestinalis* genotype		Symptomatic (n=18)		Asymptomatic (n=43)	
Assemblage(s)	Sub-assemblage(s)	No.	%	No.	%
A	A (unidentified)	0		0	
	AI	0		0	
	AII	1	5.6	4	9.3
B	B (unidentified)	5	27.6	19	44.2
	BIII	1	5.6	0	
	BIV	1	5.6	5	11.6
Mixed types	A+B	1	5.6	2	4.7
(A+B)	A+BIII	1	5.6	0	
	A+BIV	0		1	2.3
	AI+BIII	1	5.6	0	
	AI+BIV	2	11.1	0	
	AII+B	0		7	16.3
	AII+BIII	4	22.1	3	6.9
	AII+BIV	1	5.6	2	4.7

Lane M: 100-bp DNA ladder;
Lane 1 and 2: DNA-banding patterns of BIII and BIV sub-assemblages of Giardia intestinalis respectively;
RFLP=Restriction fragment length polymorphism

### Demographic and clinical implications of different sub-assemblages

Of the 61 *G. intestinalis* cyst-positive subjects, 18 presented with gastrontestinal symptoms (Table 1 and 5), i.e. five (22.7%) of 22 Bangkok residents (Group 2) and 13 (33.3%) of 39 Ratchaburi inhabitants (Group 4). Three of the five Bangkok subjects had cyst assemblage A+B, and two had assemblage B only. Seven (53.9%) symptomatic subjects of the rural residents had mixed A+B cysts, five (38.5%) had B only, and one (7.6%) had A only. Table 5 gives details on age, clinical diagnosis, and other parasites found in stools of the symptomatic subjects.

**Table 5.** Age of subjects with gastrointestinal disturbances whose stool samples contained *Giardia intestinalis* only or with other parasite(s)

Group	No. of subjects	Age (years)	Clinical diagnosis/other parasite(s) found	Assemblage(s)/sub-assemblage(s)
2	18	38	Abdominal pain, diarrhoea with soft stool*/Blastocystis hominis*	AII+BIII
(Siriraj Hospital, Bangkok)	19	50	Abdominal pain with nausea, anaemia	AII+BIV
	20	9	Diarrhoea, bronchitis	B
	21	54	Diarrhoea, chronic urticaria	B
	22	66	Diarrhoea with watery stool, hypertesion, dyslipidaemia	AI+BIV
4	49	5	Diarrhoea*/Entameoba coli*	B
(Community, Ratchaburi province)	50	3	Abdominal pain, diarrhoea with soft stool	AII+BIII
	51	7	Diarrhoea	A+B
	52	8	Abdominal pain, diarrhoea*/Endolimax nana*	A+BIII
	53	2	Abdominal pain with nausea	BIV
	54	4	Abdominal pain, diarrhoea	BIII
	55	7	Abdominal pain, diarrhoea with soft stool*/Necator americanus*	AII+BIII
	56	6	Watery diarrhoea	AI+BIII
	57	6	Diarrhoea	B
	58	50	Watery diarrhoea*/B. hominis*	AII
	59	4	Abdominal pain, diarrhoea with soft stool	AII+BIII
	60	5	Diarrhoea with watery stool	AI+BIV
	61	5	Diarrhoea*/E. histolytica, E. coli, E. nana*	B

All the three (100%) subjects with mixed AI+BIII sub-assemblages (no. 56 of Group 4) or AI+BIV sub-assemblages (no. 22 of Group 2 and 60 of Group 4) had gastrointestinal symptoms. There were six (27%) symptomatic subjects among the 22 individuals with AII sub-assemblage. These subjects were no. 18 and 19 of Group 2 and no. 50, 55, 58, and 59 of Group 4. Of these two subjects, i.e. no. 18 of Group 2, aged 38 years, and no. 55 of Group 4, aged seven years, had mixed infection of *G. intestinalis* (AII+BIII) and *Blastocystis hominis*, and AII+BIII and hookworm *(Necator americanus)* respectively. No. 19 of Group 2, aged 50 years, and no. 50, 58, and 59 of Group 4, aged 3, 7 and 4 years, respectively, had AII+BIV, AII+BIII, AII alone, and AII+BIII.

Of the 10 (45.5%) subjects who had BIII sub-assemblage cysts, seven (70%) had gastrointestinal symptoms (Table 5), i.e. no. 18 of Group 2 and no. 50, 52, 54, 55, 56, and 59 of Group 4. One subject—no. 52, aged 8 years—had a mixed infection of *G. intestinalis* with *Endolimax nana*. Of the 12 subjects whose cysts were sub-assemblage BIV, four (33%) had gastrointestinal symptoms, and their stool samples revealed only *G. intestinalis* cysts (Table 3), i.e. no. 19 (AII+BIV) and 22 (AI+BIV) of Group 2 and no. 53 (BIV) and 60 (AI+BIV) of Group 4, aged 50, 66, 2 and 4 years respectively.

Five subjects with gastrointestinal symptoms had *G. intestinalis* cysts of B assemblage only, i.e. no. 20 and 21 of Group 2 and no. 49, 52, and 61 of Group 4. No. 49 had mixed infections of *G. intestinalis* with *Entamoeba coli,* and no. 61 had *G. intestinalis* with *E. histolytica, E. coli,* and *E. nana*.

The *G. intestinalis* sub-assemblages of all the symptomatic subjects were also verified by comparing the nucleotide sequences of their gene segment amplicons with the respective sequences of the GenBank database (data not shown).

Of the five symptomatic subjects of Group 2 (Bangkok inhabitants), four (80%) were adults, i.e. no. 18, 19, 21, and 22, aged 38, 50, 54, and 66 years respectively, and only one (20 %) was a child (no. 20, aged 9 years). Of these, only no. 18 had a mixed infection of *G. intestinalis* with *B. hominis*. It can, thus, be concluded that the most (80%) cases with symptomatic giardiasis in Group 2 were adult/elderly. Of the 13 symptomatic subjects of Group 4 (from communities of Ratchaburi province), 12 (92%) were children, and only one (8%, no. 58) was an adult aged 50 years. Of these 13 subjects, five had stool samples that revealed parasites other than *G. intestinalis*, i.e. no. 49 with *E. coli,* no. 52 with *E. nana*, no. 55 with hookworm, no. 58 with *B. hominis*, and no. 61 with *E. histolytica, E. coli,* and *E. nana*.

## DISCUSSION

Most human *Giardia*-associated infections occur through ingestion of parasite cysts in contaminated water or directly through faecal-oral transmission (2, 23). The infectious dose of the parasite can be as few as 10 cysts or less (24). Thus, water sanitation and personal hygiene determine the prevalence of giardiasis in a community (25). The results of this study showed that the prevalence of giardiasis in Thailand was much lower than previously reported which was as high as 37.7% in 2003 in Pathum-thani province at the outskirts of Bangkok (17) where inhabitants had a similar lifestyle to the Ratchaburi communities of this study who had a prevalence of 4.1%, which reflects improved sanitary conditions, healthcare, and health education in the country during recent years. Moreover, the prevalence of *Giardia*-associated infections among the Bangkok residents (0.36%) was about 10 times lower than that of the rural communities (4.1%). People living in Bangkok have easier access to clean water and proper waste disposal than many rural areas where some peasants still bath and wash their dishes and clothes in canals or rivers which are easily contaminated and where the parasite may flourish. Moreover, there are more pets, poultry, livestock, and other animals running around freely in rural communities serving as potential sources of human infections due to *Giardia* (2).

The molecular characterization of the *Giardia* spp. is useful for understanding the biology of the parasite, its host preferences, epidemiology, and pathogenicity (2). However, information on *G. intestinalis* isolates of humans and animals and their genotypes in Thailand is scarce. In this study, PCR-based genotyping of *G. intestinalis* cysts was performed. The genomic DNA extracted from the cysts of the subjects were used as templates in a one-step PCR for the amplification of the 148- and 81-bp *tpi* gene segments for determining assemblage A and B respectively (19). This single-step PCR of the *tpi* gene segments for assemblage classification was simple and rapid. It was also highly sensitive (sensitivity 100%) compared to the two-step PCR performed on DNA extracted from the whole faeces, using a different set of primers, with a sensitivity of 94% for sporadic cases and 88% for samples of a nursery outbreak (26). The assemblage identification of the cysts from 61 *G. intestinalis*-infected subjects revealed that 8% (5 of 61) of the cyst preparations were assemblage A only, 51% (31 of 61) were assemblage B only, and 41% (25 of 61) were a mixture of assemblage A and B. Our findings conformed to the notion that, although the *Giardia* spp. are heterogeneous and can be classified into A-G assemblages which infect a wide range of hosts, only the assemblage A and B are contagious for humans (6, 23). Altogether, 30 individuals harboured cysts of assemblage A, or A+B mixture and 56 individuals with B or A+B cysts. The prevalence of assemblage B infection was almost twice as high as that of assemblage A infection. These data are similar to studies carried out in Bangladesh (14), the Philippines (27), India (15), England (28), the Netherlands (29), and Brazil (30) but are different from data reported from Korea (31) and Mexico (32) where assemblage A was predominant.

A further classification of the cysts into A and B sub-assemblages based on RFLP of the 384-bp β-*giardin* and 432-bp *gdh* gene segments respectively showed that cysts of only three subjects (12%) belonged to sub-assemblage AI, most (88%) belonged to sub-assemblage AII, and cysts of 10 (45.5%) and 12 (54.5%) subjects belonged to the BIII and BIV sub-assemblages respectively. Earlier reports indicated that the AI sub-assemblage and the B assemblage, regardless of the B sub-assemblages, have a broad host range, including pets, wildlife, and livestock while the AII sub-assemblage is more limited to human subjects (2, 33). Thus, it is possible that the AI and B infections found in this study were zoonotic while the AII infections were anthroponotic.

Although there was no clear-cut correlation of assemblages and sub-assemblages with symptomatic giardiasis in this study (Table 5), all the three subjects with AI sub-assemblage, either AI+BIII (no. 56, aged 6 years) or AI+BIV (no. 22, aged 66 years and no. 60, aged 5 years) had gastrointestinal symptoms. The stool sample of subject no. 18 also had *B. hominis* which is regarded as a potential pathogen and may occasionally cause abdominal pain, nausea, bloating and/or diarrhoea; therefore, the gastrointestinal symptoms experienced by this subject might have been due to the organism that co-infected. Nevertheless, the two (66%) remaining subjects who had only *G. intestinalis*-associated infection with sub-assemblage AI+BIII and AI+BIV had symptomatic giardiasis. Seventy percent of the subjects with BIII sub-assemblage, either BIII alone, AI+BIII, or AII+BIII, had gastrointestinal symptoms. Four of these seven subjects had *Giardia* cysts only, and no other pathogens were found in their stool samples while three subjects had mixed infections of *G. intestinalis* with *B. hominis* (no. 18), *E. nana* (no. 52), and hookworm (no. 55). *B. hominis* and hookworm can cause diarrhoea and other gastrointestinal disturbances while *E. nana* is non-pathogenic for humans. Thus, five of the seven subjects with sub-assemblage BIII may be regarded as symptomatic giardiasis. The *in vivo*-expressed antigens/virulence factors, the host-parasite interplay, and the host factors determining the susceptibility to *G. intestinalis* at a molecular level need further elucidation.

Only one (20%) of the five symptomatic subjects of the Bangkok group was a child (no. 20, aged 9 years) while the other four subjects were aged 66, 38, 50, and 54 years. The reverse picture was seen for the symptomatic subjects of the Ratchaburi group, i.e. only one (8%) of the 13 symptomatic subjects was elderly (no. 58, aged 50 years) while the remaining 12 subjects (92%) were children aged 2–7 years. Our findings conformed to data previously reported from other countries that symptomatic giardiasis is mainly confined to the young and the elderly (34, 35). Reasons usually given to explain the susceptibility of these age-groups are the immaturity of the immunological apparatus in the former and the (replicative) immune senescence/immune incompetence of the latter (36, 37). Nevertheless, other parasite factors and host factors and their interplay have also been suggested as contributing to the pathophysiology observed in clinical giardiasis (37). During an infection, a clone of *Giardia* spp. may diversify into a complex mixture of different antigenic types (38) and depending upon both host immunological factors, such as specific secretory IgA, and non-immunological factors, such as the intestinal protease, the *Giardia* trophozoite variant(s) that can resist the hostile environment in the intestines, would proliferate and cause morbidity (38). Moreover, *Giardia* lectin promotes adherence of the parasite to the intestinal brush border, and the parasite uses the ventral sucker disc to colonize the epithelium (38), which consequently may cause acute/chronic diarrhoea, steatorrhoea, and malabsorption and, in a prolonged infection, lead to growth retardation depending on the burden of parasite and its virulence. There is a possibility that *Giardia* produce a toxin or at least metabolic products that may contribute to their pathogenicity (39). Obviously, much more is to be learnt about this inscrutable parasitic infection. This study provides a baseline message on genotypes of the isolates of *G. intestinalis* from humans in Thailand and also demonstrates a correlation of symptomatic giardiasis with age of the host.

## ACKNOWLEDGEMENTS

The Thailand Research Fund, the Commission on Higher Education, and the National Science and Technology Development Agency supported this work. The authors thank the staff of the Central Laboratory of the Department of Parasitology, Faculty of Medicine Siriraj Hospital, Mahidol University, Bangkok, for identifying *G. intestinalis*-positive stool samples. They also thank the Children Foundation and the staff of the Health Promotion Section of Suanpung district, Ratchaburi province, for their assistance in collecting the faecal samples of volunteers. Thanks are also due to Dr. Mark Roselieb for reading the manuscript.

## References

[1] Monis PT, Caccio SM, Thompson RC (2009). Variation in *Giardia*: towards a taxonomic revision of the genus. Trends Parasitol.

[2] Adam RD (2001). Biology of *Giardia lamblia*. Clin Microbiol Rev.

[3] Thompson RC (2004). The zoonotic significance and molecular epidemiology of *Giardia* and giardiasis. Vet Parasitol.

[4] Nash TE, McCutchan T, Keitser D, Dame JB, Conrad JD, Gillin FD (1985). Restriction-endonuclease analysis of DNA from 15 *Giardia* isolates obtained from humans and animals. J Infect Dis.

[5] Monis PT, Andrews RH, Mayrhofer G, Ey PL (2003). Genetic diversity within the morphological species *Giardia intestinalis* and its relationship to host origin. Infect Genet Evol.

[6] Mayrhofer G, Andrew RH, Ey PL, Chilton NB (1995). Division of *Giardia* isolates from humans into two genetically distinct assemblages by electrophoretic analysis of enzymes encoded at 27 loci and comparison with *Giardia muris*. Parasitol.

[7] Caccio SM, Thompson RC, McLauchlin J, Smith HV (2005). Unravelling *Cryptosporidium**Giardia* epidemiology. Trends Parasitol.

[8] World Health Organization (1998). The World Health Organization report 1998—life in the 21^st^ century: a vision for all.

[9] Ortega YR, Adam RD (1997). *Giardia*: overview and update. Clin Infect Dis.

[10] Farthing MJ (1996). Giardiasis. Gastroenterol Clin North Am.

[11] Fraser DN, Bilenkoan N, El-On J, Naggan L (2000). *Giardia lamblia* carriage in Israel Bedouin infants: risk factors and consequences. Clin Infect Dis.

[12] Dib HH, Lu SQ, Wen SF (2008). Prevalence of *Giardia lamblia* with or without diarrhea in South East, South East Asia and the Far East. Parasitol Res.

[13] Mohandas K, Sehgal R, Sud A, Malla N (2002). Prevalence of intestinal parasitic pathogens in HIV-seropositive individuals in northern India. Jpn J Infect Dis.

[14] Haque R, Mondal D, Kirkpatrick BD, Akther S, Farr BM, Sack RB (2003). Epidemiologic and clinical characteristics of acute diarrhea with emphasis on *Entamoeba histolytica* infections in preschool children in an urban slum of Dhaka, Bangladesh. Am J Trop Med Hyg.

[15] Ajjampur SSR, Sankaran P, Kannan A, Sathyakumar K, Sarkar R, Gladstone BP (2009). *Giardia duodenalis* assemblages associated with diarrhea in children in South India identified by PCR-RFLP (short report). Am J Trop Med Hyg.

[16] Haque R, Mondal D, Karim A, Molla IH, Rahim A, Faruque ASG (2009). Prospective case-control study of the association between common enteric protozoal parasites and diarrhea in Bangladesh. Clin Infect Dis.

[17] Saksirisampant W, Nuchprayoon S, Wiwanitkit V, Yenthakam S, Ampavasiri A (2003). Intestinal parasitic infestations among children in an orphanage in Pathum Thani province. J Med Assoc Thai.

[18] Sornmani S, Vivatanasesth P, Bunnag T, Intarakhao C, Harinasuta C (1973). A study on pattern of socioeconomic and health status in relation to parasitic diseases in the habitants around Ubolratana dam in northeast Thailand. Southeast Asian J Trop Med Public Health.

[19] Cacciò SM, De Giacomo M, Pozio E (2002). Sequence analysis of the beta-*giardin* gene and development of a polymerase chain reaction-restriction fragment length polymorphism assay to genotype *Giardia intestinalis* cysts from human faecal samples. Int J Parasitol.

[20] Read CM, Monis PT, Thompson RC (2004). Discrimination of all genotypes of *Giardia intestinalis* at the glutamate dehydrogenase locus using PCR-RFLP. Infect Genet Evol.

[21] Bertrand I, Albertini L, Schwartzbrod J (2005). Comparison of two target genes for detection and genotyping of *Giardia lamblia* in human feces by PCR and PCR restriction fragment length polymorphism. J Clin Microbiol.

[22] Thompson JD, Gibson TJ, Plewniak F, Jeanmougin F, Higgins DG (1997). The CLUSTAL_X Windows interface: flexible strategies for multiple sequence alignment aided by quality analysis tools. Nucleic Acids Res.

[23] Flanagan PA (1992). *Giardia*: diagnosis, clinical course, and epidemiology. A review. Epidemiol Infect.

[24] Rendtorff RC (1954). The experimental transmission of human intestinal protozoan parasites. II. *Giardia lamblia* cysts given in capsules. Am J Hyg.

[25] Stuart JM, Orr HJ, Warburton FG, Jeyakhanth S, Pugh C, Morris I (2003). Risk factors for sporadic giardiasis: a case-control study in southwestern England. Emerg Infect Dis.

[26] Amar CF, Dear PH, Pedraza-Diaz S, Looker N, Linnane E, McLauchlin J (2002). Sensitive PCR-restriction fragment length polymorphism assay for detection and genotyping of *Giardia duodenalis* in human feces. J Clin Microbiol.

[27] Yason JA, Rivera WL (2007). Genotyping of *Giardia duodenalis* isolates among residents of slum area in Manila, Philippines. Parasitol Res.

[28] Amar CF, Dear PH, McLauchlin J (2003). Detection and genotyping by real-time PCR/RFLP analyses of *Giardia duodenalis* from human feces. J Med Microbiol.

[29] Van der Giessen JW, de Vries A, Roos M, Wielinga P, Kortbeek LM, Mank TG (2006). Genotyping of *Giardia* in Dutch patients and animals: a phylogenetic analysis of human and animal isolates. Int J Parasitol.

[30] Kohli A, Bushen OY, Pinkerton RC, Houpt E, Newman RD, Sears CL (2008). *Giardia duodenalis* assemblage, clinical presentation and markers of intestinal inflammation in Brazilian children. Trans R Soc Trop Med Hyg.

[31] Yong T, Han K, Yang H, Park S (2002). PCR-RFLP analysis of *Giardia intestinalis* using a *Giardia*-specific gene, GLORFC4. Parasite.

[32] Ponce-Macotela M, Martinez-Gordillo MN, Bermudez-Cruz RM, Salazar-Schettino PM, Ortega-Pierres G, Ey PL (2002). Unusual prevalence of the *Giardia intestinalis* A-II subtype amongst isolates from humans and domestic animals in Mexico. Int J Parasitol.

[33] Thompson RCA, Palmer SR, Soulsby EJL, Simpson DIH (1998). *Giardia* infection. Zoonosis, biology, clinical practice and public health control.

[34] Fraser D, Dagan R, Naggan L, Greene V, El-On J, Abu-Rbiah Y (1997). Natural history of *Giardia lamblia**Cryptosporidium* infections in a cohort of Israeli Bedouin infants: a study of a population in transition. Am J Trop Med Hyg.

[35] Sahagun J, Clavel A, Goñi P, Seral C, Llorente MT, Castillo FJ (2008). Correlation between the presence of symptoms and the *Giardia intestinalis* genotype. Eur J Clin Microbiol Infect Dis.

[36] Gorozynski RM, Terzioglu E (2008). Aging and the immune system. Int Urol Nephrol.

[37] Katelaris PH, Farthing MJ (1992). Diarrhea and malabsorption in giardiasis: a multifactorial process. Gut.

[38] Muller N, Gottstein B (1998). Antigenic variation and the murine immune response to *Giardia lamblia*. Int J Parasitol.

[39] Nash TE, Gallin FD, Smith PD (1983). Excretory-secretory products of *Giardia lamblia*. J Immunol.

